# Endemic Foci of the Tick-Borne Relapsing Fever Spirochete *Borrelia crocidurae* in Mali, West Africa, and the Potential for Human Infection

**DOI:** 10.1371/journal.pntd.0001924

**Published:** 2012-11-29

**Authors:** Tom G. Schwan, Jennifer M. Anderson, Job E. Lopez, Robert J. Fischer, Sandra J. Raffel, Brandi N. McCoy, David Safronetz, Nafomon Sogoba, Ousmane Maïga, Sékou F. Traoré

**Affiliations:** 1 Laboratory of Zoonotic Pathogens, Rocky Mountain Laboratories, National Institute of Allergy and Infectious Diseases, Hamilton, Montana, United States of America; 2 Laboratory of Malaria and Vector Research, National Institute of Allergy and Infectious Diseases, Twinbrook, Maryland, United States of America; 3 Laboratory of Virology, Rocky Mountain Laboratories, National Institute of Allergy and Infectious Diseases, Hamilton, Montana, United States of America; 4 Malaria Research and Training Center, University of Sciences, Techniques and Technologies of Bamako, Bamako, Mali; University of California Davis, United States of America

## Abstract

**Background:**

Tick-borne relapsing fever spirochetes are maintained in endemic foci that involve a diversity of small mammals and argasid ticks in the genus *Ornithodoros*. Most epidemiological studies of tick-borne relapsing fever in West Africa caused by *Borrelia crocidurae* have been conducted in Senegal. The risk for humans to acquire relapsing fever in Mali is uncertain, as only a few human cases have been identified. Given the high incidence of malaria in Mali, and the potential to confuse the clinical diagnosis of these two diseases, we initiated studies to determine if there were endemic foci of relapsing fever spirochetes that could pose a risk for human infection.

**Methodology/Principal Findings:**

We investigated 20 villages across southern Mali for the presence of relapsing fever spirochetes. Small mammals were captured, thin blood smears were examined microscopically for spirochetes, and serum samples were tested for antibodies to relapsing fever spirochetes. *Ornithodoros sonrai* ticks were collected and examined for spirochetal infection. In total, 11.0% of the 663 rodents and 14.3% of the 63 shrews tested were seropositive and 2.2% of the animals had active spirochete infections when captured. In the Bandiagara region, the prevalence of infection was higher with 35% of the animals seropositive and 10% infected. Here also *Ornithodoros sonrai* were abundant and 17.3% of 278 individual ticks tested were infected with *Borrelia crocidurae*. Fifteen isolates of *B. crocidurae* were established and characterized by multi-locus sequence typing.

**Conclusions/Significance:**

The potential for human tick-borne relapsing fever exists in many areas of southern Mali.

## Author Summary

Tick-borne relapsing fever is a spirochete-caused, recurrent illness acquired by the bite of fast-feeding ticks. In Mali, the potential for people to acquire relapsing fever is unknown although a few human cases have been reported there. Human malaria is also abundant in Mali, and could be complicating the diagnosis of relapsing fever. The relapsing fever spirochete, *Borrelia crocidurae*, is maintained in natural cycles involving small mammals and its tick vector *Ornithodoros sonrai*. Therefore, we investigated 20 villages across southern Mali to determine if relapsing fever spirochetes were circulating in small mammals and ticks that lived with people. We found that 11.3% of the 726 mammals tested showed evidence of prior infection, while 2.2% of the animals were actively infected. The tick vector was abundant in two villages we sampled, and overall 17.3% of the individual ticks tested were infected with spirochetes. We also isolated the spirochetes, *Borrelia crocidurae*, from rodents and ticks and compared their genetic makeup to other species of African spirochetes. We conclude that in some areas of Mali, people are at risk of acquiring tick-borne relapsing fever. Therefore, we recommend that blood smears from acutely ill patients be examined microscopically for spirochetes.

## Introduction

The epidemiology of tick-borne relapsing fever was founded on the works of several independent investigators working across central Africa during the first decade of the 20^th^ century. David Livingstone is credited with the first written account in 1857 of a malady associated with the bite of soft ticks in areas now known as Angola and Mozambique [1], although the identity and route of transmission of the etiological agent were not discovered for another 45 years. Then in several closely dated publications of clinical and field observations, spirochetes were reported in the blood of acutely ill patients [2]–[6], and the soft tick *Ornithodoros moubata* was identified as the vector, transmitting the bacteria when these naturally infected ticks were fed experimentally on cercopithecus monkeys [3], [7]–[9]. The seminal work was done by J. Everett Dutton and John Todd while working in the eastern region of the Congo Free State (now the Democratic Republic of Congo) [10]. Both men contracted the infection while performing autopsies, and Dutton died there on February 27, 1905 [3]. One year later, the spirochete that caused tick-borne relapsing fever across central Africa was named *Spirillum Duttoni* [11], now named *Borrelia duttonii* [12], to honor Dutton's contributions and sacrifice while working on tick fever and other tropical diseases including malaria and trypanosomiasis.

*Borrelia duttonii* has no known nonhuman animal reservoir, although many investigations have tried to demonstrate that such associations with wild and domestic animals exist. This spirochete is transmitted from person to person by the bite or coxal fluid of *O. moubata* [13]. However, all other species of tick-borne relapsing fever spirochetes are maintained in enzootic foci that involve a diversity of small mammals [14]. The first spirochete reported in a wild African mammal was *Spirochaeta crocidurae*, which was found in a shrew *Crocidura stampflii* in Dakar, Senegal [15]. This spirochete, now named *Borrelia crocidurae*, is likely widespread across much of Africa north of the equator, from Egypt to Senegal and north to Tunisia [16]–[18]. The spirochete infects a variety of wild and peridomestic rodents and shrews, and is transmitted by two species of soft ticks, *Ornithodoros erraticus* and *Ornithodoros sonrai*. These ticks were previously considered two varieties, the large and small form, respectively, of *O. erraticus* [19]. Sautet and Witkowski [20] named the small form *O. sonrai*, in honor of the ancient Sonrai Empire centered at Gao, Mali, from where the ticks were described. This species of tick is the primary vector of *B. crocidurae* in sub-Saharan Africa [21], [22].

Most ecological and epidemiological studies of tick-borne relapsing fever caused by *B. crocidurae* in West Africa have been done in Senegal. In 1989, a series of investigations were begun soon after a French child living there contracted a recurrent febrile illness that was originally thought to be malaria, but after three months and seven acute episodes the illness was diagnosed as a borrelia infection [23]. Tick-borne relapsing fever of humans is prevalent in Senegal, and *O. sonrai* and *B. crocidurae* are associated with numerous species of small mammals in many regions of the country [18], [22]–[26].

The potential risk for humans to acquire relapsing fever infection in Mali immediately to the east of Senegal is not well known, and only a few human cases have been reported from there. Some forays into Mali by Senegalese-based investigators found *O. sonrai* in the burrows of small mammals, and some of the ticks were infected with *B. crocidurae* based on PCR and detection of spirochetal DNA [18], [21], [27]; however, previous efforts directed at tick-borne relapsing fever in Mali are not clear. Rodhain et al. [28] identified two human cases in southwestern Mali in 1977 and 1988, and two more recent human cases were diagnosed in France soon after the patients arrived there from Mali where they had become infected [29], [30]. Clearly, tick-borne relapsing fever has occurred in Mali and the illness may be confused with malaria, as was suspected in Togo [31]. As stated in Manson's Tropical Diseases for the diagnosis of relapsing fever: “This fever is most usually confounded with subtertian malaria, from which it may be indistinguishable on clinical grounds” [32]. Therefore, given the high incidence of malaria in Mali [33], and the lack of information regarding the prevalence of tick-borne relapsing fever there, we initiated studies to determine if there were endemic foci that involved small mammals, ticks and spirochetes. Here we identify several areas with evidence of infection, and discuss one region in particular that has a high prevalence of infected small mammals and ticks that live in close association with humans. In these villages in south central Mali, the potential risk for humans to acquire tick-borne relapsing fever is significant.

## Methods

### Ethics statement

The Rocky Mountain Laboratories, NIAID, NIH, Animal Care and Use Committee approved study protocols #2008-1 and #2011-48 to perform the animal field studies, and protocols #2009-32 and #2009-87 for the feeding of ticks, mouse infection and isolation of relapsing fever spirochetes. All work in our study was conducted adhering to the institution's guidelines for animal husbandry, and followed the guidelines and basic principals in the United States Public Health Service Policy on Humane Care and Use of Laboratory Animals, and the Guide for the Care and Use of Laboratory Animals. Residents in the villages gave informed consent prior to our setting traps and collecting ticks in their houses.

### Mammal sampling

**Figure 1:**
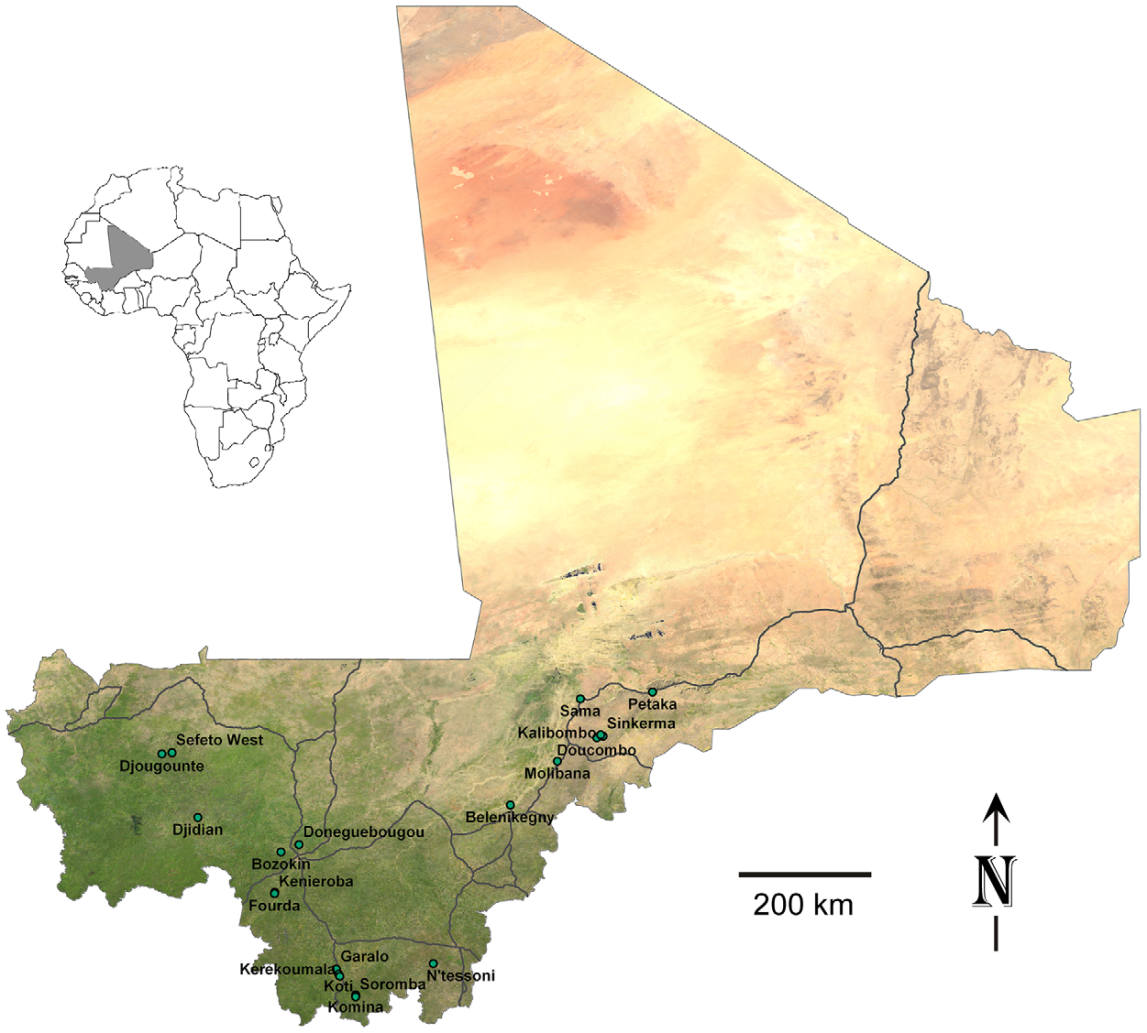
Map of Mali with the names and locations of the 20 villages investigated. The location of Mali in the African continent is identified in grey.

We collected small mammals in 20 villages across southern Mali from December 2007 to October 2011. The locations varied from latitude 10° 35′ 20.8″ to 15° 01′ 25.3″ N and longitude 2° 50′ 55.0″ to 9° 58′ 32.5″ W (Table 1) (Figure 1). The areas sampled ranged from the drier Sahel in the north to the moister wooded savannah in the south. The small mammals were captured alive in Sherman live traps (H. B. Sherman Traps, Tallahassee, FL). During the first two field efforts (December 2007 and January 2009), we used both small and large traps: small trap size was 5.2×6.4×16.5 cm; large trap size was 7.6×8.9×22.8 cm. However, the larger traps were much more productive at capturing animals and thereafter we used only them. Traps were set in the late afternoon with bait comprised of locally acquired crushed peanuts, chopped onions and occasionally pieces of dried fish. Traps were placed inside and outside houses and collected early the next morning, at which time the animals were processed. The outside location of traps varied among the villages from immediately adjacent to the walls of houses to community gardens on the outskirts of the village. The animals were euthanized by the inhalation of isoflurane, and a terminal blood sample was collected via intracardiac puncture with a 1 ml tuberculin syringe and a 26-gauge 3/8-inch needle. Thin blood smears were made on glass microscope slides. The animals were examined for ectoparasites, which if found were collected in 70% ethanol.

**Table 1:** Villages sampled, their administrative district and number, and geographic coordinates.

Village	District/No.	Latitude (North)	Longitude (West)
Sefeto West	Kayes/I	14° 08′ 25.8″	09° 49′ 37.2″
Djougounte	Kayes/I	14° 07′ 19.2″	09° 58′ 32.5″
Djidian	Kayes/I	13° 12′ 02.5″	09° 27′ 13.7″
Bozokin	Koulikoro/II	12° 41′ 53.2″	08° 14′ 35.2″
Kenieroba	Koulikoro/II	12° 06′ 43.9″	08° 19′ 55.9″
Fourda	Koulikoro/II	12° 05′ 29.4″	08° 20′ 06.0″
Doneguebougou	Koulikoro/II	12° 48′ 18.0″	07° 58′ 49.1″
N'Tessoni	Sikasso/III	11° 04′ 19.2″	06° 01′ 37.2″
Soromba	Sikasso/III	10° 35′ 20.8″	07° 09′ 20.5″
Komina	Sikasso/III	10° 36′ 58.7″	07° 09′ 29.9″
Kerekoumala	Sikasso/III	10° 53′ 22.2″	07° 23′ 15.7″
Garalo	Sikasso/III	10° 59′ 36.6″	07° 26′ 13.9″
Kotie'	Sikasso/III	10° 55′ 03.4″	07° 24′ 20.5″
Belenikegny	Ségou/IV	13° 22′ 56.6″	04° 55′ 00.1″
Molibana	Mopti/V	14° 00′ 55.8″	04° 13′ 51.6″
Sama	Mopti/V	14° 55′ 24.6″	03° 53′ 49.9″
Sinkerma	Mopti/V	14° 22′ 50.9″	03° 34′ 05.5″
Petaka	Mopti/V	15° 01′ 25.3″	02° 50′ 55.0″
Kalibombo	Mopti/V	14° 24′ 01.4″	03° 36′ 01.8″
Doucombo	Mopti/V	14° 21′ 18.7″	03° 39′ 26.3″

### Identification of mammals

The animals were tentatively identified to genus or species in the field based on external characters [34]–[36]. Their body weight was measured in grams with a Pesola spring scale (PESOLA AG, Baar, Switzerland), gender determined, and lengths of the head & body and tail were measured in centimeters. Each animal was photographed with a digital camera for future reference. One of us (TGS) visited the Smithsonian Institution's African mammal collection to examine specimens collected from various locations in West Africa to examine the external characters and skulls to assist in the identifications. Skulls from 60 animals collected in Mali were prepared for museum voucher specimens following standard curatorial procedures [37] and compared to illustrations and keys [36]. The nomenclature and taxonomic status of the animals were based on currently accepted names [38], [39].

One external ear pinna was collected from every animal and preserved in 70% ethanol for DNA extraction and molecular identification of the species. A 3-mm round skin punch biopsy was extracted later from each external ear sample and DNA was purified with the DNeasy Blood and Tissue Kit, 96-well format (QIAGEN Sciences, Inc., Germantown, MD) following the manufacturer's instructions. The mitochondrial (mt) cytochrome b (*cyt-b*) DNA was amplified with the PCR primers L14723 and H15915 [40] (Table 2) and the Go Taq Flexi DNA Polymerase kit (Promega Corp., Madison, WI) with an initial denaturation at 96°C for 3 min, followed by 35 cycles of 94°C for 30 sec, 55°C for 30 sec, 72°C for 2.5 min, and a final heating at 72°C for 7 min. PCR products were purified with the QIAquick PCR Purification Kit (QIAGEN Sciences) following the manufacturer's Spin Protocol. DNA sequences of the amplicons were produced with the BigDye Terminator v3.1 Cycle Sequencing Kit (Applied Biosystems, Foster City, CA) with reactions run for 45 cycles of 95°C for 10 sec, 50°C for 5 sec, and 60°C for 4 min. The sequence reaction products were cleaned with the BigDye XTerminator Purification Kit (Applied Biosystems) and sequenced with an Applied Biosystem's 3730xl DNA Sequencer. These sequences were submitted to GenBank at the NCBI using BLASTn [41] to determine the identification of each individual. GenBank accession numbers representative for each species we captured are in the results.

**Table 2:** Gene targets and primer sequences used in the study.

Target and Primers	Sequence (5′ to 3′)	Reference
Mammal		
*cyt-b*		
L14723	**ACCAATGACATGAAAAATCATCGT**	[40]
H15915	**TCTCCATTTCTGGTTTACAAGAC**	[40]
Tick		
mt 16S rDNA	(partial, 3′ end)	
Tick16S+1	**CTGCTCAATGATTTTTTAAATTGCTGTGG**	[80]
Tick16S−1	**CCGGTCTGAACTCAGATCAAGT**	[80]
Tick16S+2	TTGGGCAAGAAGACCCTATGAA	[80]
Tick16S−2	TTACGCTGTTATCCCTAGAG	[80]
Tick16S+3	ATACTCTAGGGATAACAGCGT	[80]
Tick16S−3	AAATTCATAGGGTCTTCTTGTC	[80]
Borrelia		
IGS		
IGS-F	**GTATGTTTAGTGAGGGGGGTG**	[81]
IGS-R	**GGATCATAGCTCAGGTGGTTAG**	[81]
IGS-Fn	AGGGGGGTGAAGTCGTAACAAG	[81]
IGS-Rn	GTCTGATAAACCTGAGGTCGGA	[81]
*glpQ*		
flank5′	**GGTATGCTTATTGGTCTTC**	[42]
BRR2	**GTTGCTCCTCCGCCAATTATTATTAAGTC**	[42]
Q1:	TTATAGCTCACAGAGGT	This Report
SPR1	GCACAGGTAGGAATGTTGGAATTTATCCTG	[42]
glpQ F-1	CAATTTTAGATATGTCTTTACCTTGTTGTTTATGCC	[42]
*flaB*		
Fla ans 5′	**TGTGATATCCTTTTAAAGAGACAAATGG**	[76]
Fla alt 3′	**TCTAAGCAATGACAATACATATTGAGG**	[76]
FlaLL	ACATATTCAGATGCAGACAGAGGT	[82]
FlaRL	GCAATCATAGCCATTGCAGATTGT	[82]
FlaLS	ACAGCTGAAGAGCTTGGAATG	[82]
FlaRS	TTTGATCACTTATCATTCTAATAGC	[82]
16S rDNA		
16RnaR	**GTATTCACCGTATCATTCTGATATAC**	[82]
16RnaL	**CTGGCAGTGCGTCTTAAGCA**	[82]
16S+	TACAGGTGCTGCATGGTTGTCG	[76]
16S−	TAGAAGTTCGCCTTCGCCTCTG	[76]
Rec9	TCGTCTGAGTCCCCATCT	[76]
Rec4	ATGCTAGAAACTGCATGA	[76]

Primers in bold were used for the initial PCR amplification; all primers were used for sequencing.

### Examination of blood

Thin blood smears were fixed with 100% methanol and stained with the *QUICK III* statpak kit (Astral Diagnostics Inc., West Deptford, NJ). Fifty fields on each slide were examined for stained spirochetes with a Nikon Eclipse E800 microscope (Nikon Instruments Inc., Melville, NY) at 600× magnification and oil immersion objective lens.

### Serology

Serum samples from most of the animals captured were tested by immunoblot for antibodies to relapsing fever spirochetes. Briefly, whole-cell lysates of *Borrelia duttonii* CR2A or *B. crocidurae* DOU (isolated during this study), and purified heterologous GlpQ from *Borrelia recurrentis* were prepared as described [42], [43] and electrophoresed in adjacent lanes by SDS-PAGE in Novex 12% Tris-Glycine Mini Gels-1 mm (Life Technologies, Carlsbad, CA). The proteins were transferred onto nitrocellulose using the iBlot Dry Blotting System and iBlot Transfer Stacks (Life Technologies) or the Mini-PROTEAN II Cell and 0.45 µm membrane (BioRad, Life Sciences Research, Hercules, CA). Each serum sample was tested at 1∶100 dilution (two low-volume samples were diluted 1∶200) with the two-lane nitrocellulose panel. Bound primary antibodies were detected with HRP-rec Protein A (Life Technologies) and the Amersham ECL Western Blotting Detection Reagents (GE Healthcare, Piscataway, NJ) or the SuperSignal West Pico Chemiluminescent Substrate (Thermo Scientific, Rockford, IL). Chemiluminescence of bound antibodies was detected with Amersham Hyperfilm ECL (GE Healthcare Ltd., Buckinghamshire, UK). A sample was considered positive if it contained antibodies reactive to 5 or more proteins in the whole-cell lysate and to the purified GlpQ protein.

### Tick collecting and identification

Attempts to collect the argasid tick vector, *Ornithodoros sonrai*, from small mammal burrows were done with a Craftsman, Incredi-Pull, 4-Cycle Blower/Vacuum 29 cc (Sears, Roebuck and Co., Hoffman Estates, IL). A tube was attached to the air intake aperture and connected to a hose (modified from a version first described by Butler et al.) [44]. One end of the hose was attached to the mouth of the intake, while the other end was inserted into the opening of a mammal burrow within the peoples' houses. A fine mesh screen was inserted into the air pathway to capture material that was aspirated into the hose. This burrow material was then removed from the device, screened again by hand and examined for live ticks.

Some ticks were preserved in 70% ethanol while others were kept alive, and all specimens were taken to the laboratory and examined with a Zeiss Stemi SV 11 Stereomicroscope (Carl Zeiss MicroImaging, Inc., Thornwood NY), and identified to species [20], [45], stage and sex. DNA samples from 208 individual ticks preserved in ethanol were purified with the DNeasy Blood and Tissue Kit, following the manufacturer's Mini Spin Column Protocol (QIAGEN Sciences). Prior to the extractions, each tick was placed in a 1.5 ml microfuge tube with the pestle and frozen together in liquid nitrogen to increase the efficiency for grinding the ticks into powder. The purified DNA was used as template for PCR that targeted the tick mt 16S rDNA and several borrelia genes (see below). The tick mt 16S rDNA was amplified and both strands were sequenced using the primers listed (Table 2) and the methods described above for the mammalian *cyt-b* gene. The chromatograms for each sequence were aligned to assure the correct identification of each base and ambiguous sequences at the ends were deleted.

### Tick feeding and isolation of spirochetes

Live ticks were fed on laboratory mice, *Mus musculus*. For this procedure, the mice were anesthetized with pentobarbital (0.5 mg/10 g body wt) via an intraperitoneal injection, the hair on the abdomen of the mouse was cut with electric clippers, and the ticks were placed on the abdomen and allowed to feed. Ticks typically took 30 to 60 minutes to feed, after which time they were placed into ventilated plastic tubes and kept within jars at 28°C and a saturated solution of KCl to maintain a relative humidity of 85% [46]. Those mice that were fed upon by ticks were caged individually and examined for spirochete infections for up to 10 days after the ticks had fed. Mice were examined for infection by nicking the tip of the tail and expressing a drop of blood (approximately 5 µl) from the tail vein onto a microscope slide, placing a cover slip over the blood, and examining the fresh sample with a Nikon Eclipse E200 dark-field microscope (Nikon Instruments Inc.) at 400× magnification. When spirochetes were detected, a terminal blood sample was collected from the anesthetized mouse via intracardiac puncture and 50–100 µl were inoculated into 5 ml of BSK-H medium (Sigma-Aldrich Co., St. Louis, MO) or mBSK-c medium [47] supplemented with 12% rabbit serum. Cultures were held at 33–35°C and examined every few days for viable spirochetes. Cultures that grew successfully were passed into 100 ml of medium and allowed to grow to late exponential phase. Aliquots of the cultures were frozen with 10%/volume dimethyl sulfoxide (DMSO) at −80°C, and DNA was purified from the remainder of the sample as described previously [48].

### Genetic analysis of spirochetes

Genomic DNA samples were analyzed by gel electrophoresis and multilocus sequence typing (MLST). Intact plasmids were resolved from chromosomal DNA in 1% agarose reverse-field gels [49] and 0.35% agarose 2-dimension gels [50] to identify linear and circular molecules. Four borrelia chromosomal loci were targeted by PCR to amplify DNA for sequencing: the intergenic spacer (IGS) between the 16S rDNA and 23S rDNA, the 16S rDNA, the flagellin *flaB* gene, and the glycerophosphodiester phosphodiesterase gene (*glpQ*) (primers and references in Table 2). The methods and parameters for PCR and sequencing were as described above for the *cyt-b* gene. The nucleotide sequences were submitted to NCBI using BLASTn for comparison to homologous sequences in the database. The new sequences were analyzed with those sequences retrieved from GenBank using the MacVector 10.1 software package (Accelrys, San Diego, CA) and multiple sequence alignments were done with CLUSTAL W [51]. Phylograms were constructed with several algorithms but those presented were built with the UPGMA and Jukes-Cantor methods provided in the MacVector package.

## Results

### Mammals captured

We set traps on 36 nights during the study in 20 villages with a total effort of 2,909 trap-nights. We captured 744 animals for an overall trap success of 26% that included 14 species of rodents and shrews; however, seven species comprised 96% (717 of 744 total) of the individuals (Table 3). *Mastomys natalensis*, *Mastomys erythroleucus*, and *Mastomys huberti* together comprised 76% of all captures (565 of 744 total). *Mastomys natalensis* was captured most frequently and this animal was the most ubiquitous, as we captured these rats at 17 of the 20 villages we sampled (Table S1). *Praomys daltoni* and the shrew, *Crocidura olivieri*, were also captured in many of the villages. In contrast, *M. huberti*, which is restricted to the Inland Delta of the Niger and Bani Rivers, was captured only in and near Belenikegny. In Soromba and the other nearby villages in the southern-most region we worked, *M. natalensis* was the only species we captured.

**Table 3:** The species and number of small mammals captured in Mali.

Species	Total	Caught Inside	Caught Outside
*Mastomys natalensis*	430	414 (96%)	16 (4%)
*Mastomys erythroleucus*	80	5 (6%)	75 (94%)
*Mastomys huberti*	55	1 (2%)	54 (98%)
*Crocidura olivieri*	57	42 (74%)	15 (26%)
*Praomys daltoni*	50	30 (60%)	20 (40%)
*Rattus rattus*	29	28 (97%)	1 (3%)
*Arvicanthis niloticus*	16	1 (6%)	15 (94%)
*Taterillus gracilis*	7	0	7 (100%)
*Acomys airensis*	5	2 (40%)	3 (60%)
*Mus musculoides*	4	0	4 (100%)
*Gerbillus campestris*	1	0	1 (100%)
*Crocidura viaria*	6	0	6 (100%)
*Crocidura fulvastra*	3	0	3 (100%)
*Crocidura* sp.	1	1 (100%)	0
	**744**	**524 (70%)**	**220 (30%)**

Individuals of some species were trapped much more frequently within houses than outside (Table 3). While most of the *M. natalensis* were captured indoors, the opposite was true for *M. erythroleucus* and *M. huberti*. The cosmopolitan roof rat, *Rattus rattus*, was found mostly in Belenikegny and all but one of these animals was captured indoors. *Crocidura olivieri* was also captured more frequently within houses than outside.

Sixty skulls were prepared from nine of the species captured (Table S2) and these samples were deposited as voucher specimens in the Smithsonian Institution, National Museum of Natural History, Division of Mammals, Collection of African Mammals, Washington DC. The specimens currently have RML numbers ranging from M#-168 to M#-568 and are awaiting museum accession numbers. Identifications of 717 of the 744 animals captured were also supported by mitochondrial *cyt-b* DNA sequences, and 36 representative sequences for 12 species are deposited in GenBank with accession numbers JX292860–JX292895 (see Table S2 for accession numbers associated with voucher skull specimens).

### Ectoparasites

No *O. sonrai* were found on any of the small mammals, which was not surprising given the short feeding time and nidicolous nature of these argasid ticks. Numerous fleas (*Xenopsylla* species) and mesostigmatid mites were collected, as were a few ixodid ticks and sucking lice. None of these arthropods are germane to the present study and will not be discussed further.

### Serological testing for antibodies

**Figure 2:**
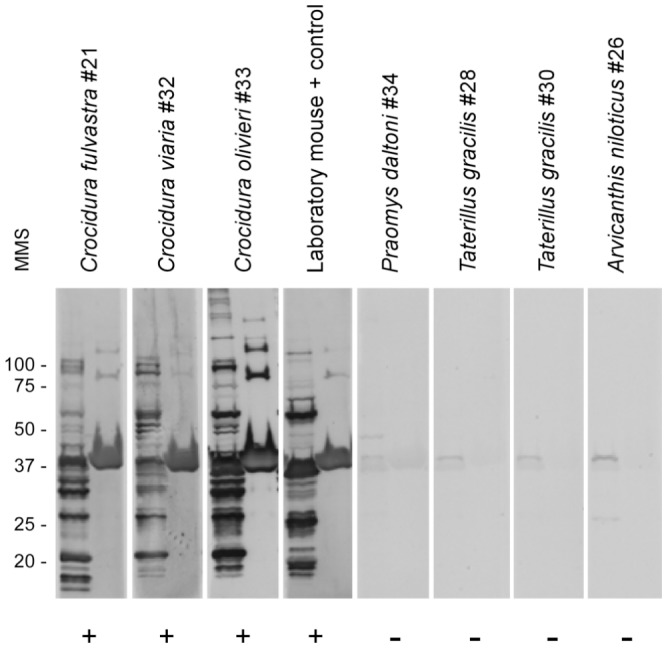
Immunoblots of selected serum samples from Malian small mammals. Representative samples are shown for positive and negative results for antibodies to relapsing fever spirochetes. The animal species and number are shown above with one experimentally infected laboratory mouse included as a positive control. Each panel has the borrelia whole-cell lysate on the left and purified GlpQ on the right. Molecular mass standards (MMS) are shown on the far left in kilodaltons. + = positive; − = negative.

Serum samples from 726 animals were tested for antibodies to investigate prior infection with relapsing fever spirochetes. For all locations, 82 animals (11.3%) were seropositive by immunoblot analysis with antibodies binding to multiple proteins in the borrelia whole-cell lysate and to the purified GlpQ (Table 4) (Figure 2). One or more of the animals captured in 14 of the 20 villages were seropositive (Table 5) (Table S1). Animals that contained antibodies to relapsing fever spirochetes were distributed from Djougounte to Petaka, the most westward and eastward locations, respectively, that we sampled. However, more than half of the seropositive animals were captured in two villages near Bandiagara: Kalibombo and Doucombo. Here, 45 of 128 of the animals (35%) were seropositive, and included *M. natalensis*, *P. daltoni* and *C. olivieri*. These three species that showed evidence of prior infection were captured in houses and lived in close proximity to humans.

**Table 4:** Small mammals tested for anti-relapsing fever spirochete antibodies.

Species	No. Tested	No. Positive (%)
*Mastomys natalensis*	422	47 (11.1)
*Mastomys erythroleucus*	78	12 (15.4)
*Mastomys huberti*	54	5 (9.3)
*Praomys daltoni*	49	8 (16.3)
*Crocidura olivieri*	53	7 (13.2)
*Crocidura viaria*	6	1 (16.7)
*Crocidura fulvastra*	3	1 (33.3)
*Arvicanthis niloticus*	16	1 (6.3)
*Rattus rattus*	29	0
*Taterillus gracilis*	7	0
*Acomys airensis*	5	0
*Mus musculoides*	2	0
*Gerbillus campestris*	1	0
*Crocidura* sp.	1	0
**Total**	**726**	**82 (11.3)**

**Table 5:** The number of mammals captured at each village, the number of serological tests performed, and the number and percent of animals seropositive.

Village	No. Captures	No. Tests	No. Positive	% Positive
Molibana	24	23	6	26.1%
Sama	6	6	3	50.0%
Sefeto West	3	3	1	33.3%
Djougounte	9	9	2	22.2%
Kalibombo	54	52	13	25.0%
Doucombo	84	76	32	42.1%
Kenieroba	20	18	1	5.6%
Fourda	17	17	1	5.9%
Sinkerma	16	16	3	18.8%
Petaka	39	39	1	2.6%
Belenikegny	198	195	14	7.2%
Soromba	46	46	2	4.3%
Doneguebougou	94	94	1	1.1%
Komina	12	12	2	16.7%
Bozokin	19	19	0	0
N'Tessoni	25	23	0	0
Kerekoumala	26	26	0	0
Garalo	22	22	0	0
Kotie'	11	11	0	0
Djidian	19	19	0	0
**Totals**	**744**	**726**	**82**	**11.3%**

### Microscopic analysis of blood

**Figure 3:**
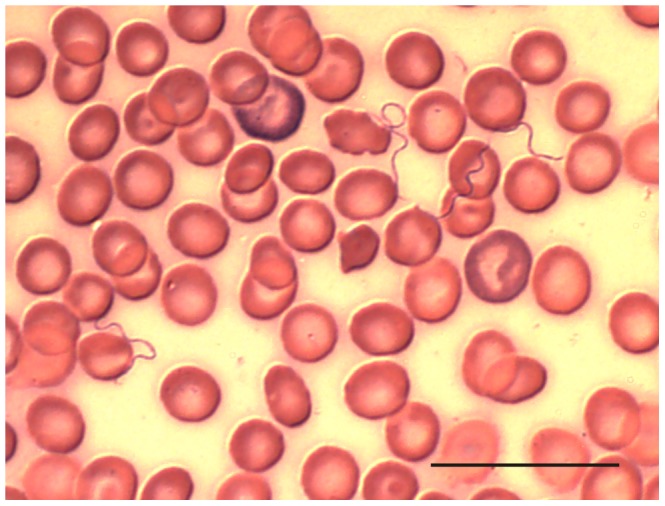
A thin blood smear showing borrelia spirochetes. This sample is from *Mastomys natalensis* #649 captured September 30, 2011, in Doucombo, Mali. Scale bar represents 20 µm.

Thin blood smears were stained and examined for spirochetes from 724 of 744 animals captured. We detected spirochetes in 16 animals (2 shrews and 14 rodents (Table 6) (Figure 3); actively infected animals were captured in five villages. The number of spirochetes observed varied from 1 to 264 spirochetes in the 50 microscopic fields examined. The majority of infected animals were captured in Kalibombo and Doucombo, the same two villages with the highest prevalence of seropositive animals. In these two villages, 13 of 130 animals (10%) had detectable spirochetes when captured, including *M. natalensis* (11 individuals), *P. daltoni* (1 individual) and *C. olivieri* (1 individual). Serum samples from three slide-positive *M. natalensis* contained live spirochetes and these samples were inoculated into laboratory mice. We isolated spirochetes from two of the mice (DOU-686 and DOU-690) and characterized them by multi-locus sequence typing (see below).

**Table 6:** Location, identification number and species of small mammal with spirochetes observed in a thin blood smear.

Village	Animal ID#	Species	No. Spirochetes^a^
Molibana	7	*Mastomys erythroleucus*	2
Djougounte	45	*Crocidura fulvastra*	2
Kenieroba	90	*Mastomys natalensis*	1
Kalibombo	517	*Crocidura olivieri*	6
Kalibombo	532	*Mastomys natalensis*	112
Doucombo	541	*Mastomys natalensis*	23
Doucombo	556	*Mastomys natalensis*	81
Doucombo	629	*Mastomys natalensis*	2
Doucombo	632	*Mastomys natalensis*	12
Doucombo	639	*Mastomys natalensis*	33
Doucombo	641	*Praomys daltoni*	47
Doucombo	646	*Mastomys natalensis*	5
Doucombo	649	*Mastomys natalensis*	264
Doucombo	686^b^	*Mastomys natalensis*	2
Doucombo	687	*Mastomys natalensis*	1
Doucombo	690^b^	*Mastomys natalensis*	140

a Number of spirochetes counted in 50 microscope fields at 600× magnification.

b *Borrelia crocidurae* was isolated in culture.

### Spirochete infection in ticks

We focused our tick collecting efforts in Doucombo and Kalibombo because of the higher percentage of infected and seropositive animals there compared to other locations. During April 2011, September–October 2011, and January 2012, we found *O. sonrai* ticks in small mammal burrows inside 24 houses in Doucombo and 14 houses in Kalibombo. In total, we collected 734 *O. sonrai*, which included 501 nymphs, 146 males and 87 females. From this total, the 208 ticks collected during April 2011 were preserved in 70% ethanol and examined individually by PCR for spirochete infection by targeting only the IGS locus. The mitochondrial 16S rDNA sequence was also determined for five of these ticks, which confirmed their identity as *O. sonrai* (GenBank accession numbers JX292854–JX292859). In this group of 208 ticks, borrelia DNA was detected in 37 (17.8%) of them (21 nymphs, 10 males, 6 females). DNA sequences for the IGS locus were of two types with 99.2% identity. The two sequences were submitted to GenBank for comparisons to other available sequences and both sequences aligned closest to the IGS sequence of the Achema strain of *B. crocidurae* that originated from *O. sonrai* ticks collected in Mauritania [52].

Ticks collected during September–October 2011 and January 2012 were kept alive and shipped to the Rocky Mountain Laboratories, NIAID, NIH, Hamilton, Montana (CDC Permit #2011-08-41 and USDA Veterinary Permit #117004). Five pools of ticks (2–11 ticks per pool) and 70 single ticks (18 nymphs, 28 males, 24 females) were fed on individual mice. Two tick pools and 11 individual ticks (2 nymphs, 7 males, 2 females) transmitted spirochetes and infected blood from these mice produced 13 isolates of spirochetes in mBSK-c medium.

### Genomic analysis of spirochetes

**Figure 4:**
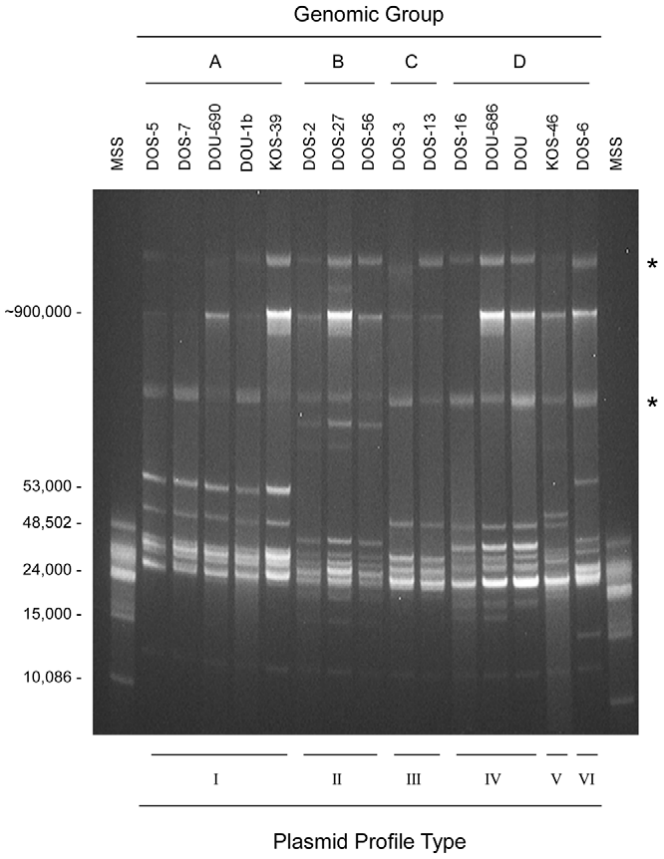
Agarose gel showing plasmid content of 15 isolates of *Borrelia crocidurae* from Mali. The isolate designations are shown above with the genomic groups determined by MLST analysis (genomic groups A–D). Stars on right are aligned with presumptive circular plasmids identified in 2-dimensional agarose gels (not shown). The plasmid types (I–VI) are on the bottom. Molecular size standards (MSS) are shown on left in base pairs.

DNA samples from the 15 isolates that originated from ticks and *M. natalensis* were analyzed by reverse field and 2-dimensional gel electrophoresis and MLST. Six distinct plasmid profiles (I–VI) were found among the undigested borrelia DNA samples from the isolates (Figure 4). Like all borreliae, these spirochetes contained numerous linear plasmids (at least ten) that ranged in size from approximately 12.5 to 100 kilobases and circular plasmids of undetermined size. The differences in the plasmid profiles for the isolates grouped them closely with the DNA sequence data presented below.

MLST segregated the 15 spirochete isolates into four primary groups (A–D) (Table 7). Each group had identical 16S rDNA and *flaB* sequences that were unique from members of the other groups, while *glpQ* sequences segregated the spirochetes into the same groups for 13 of the 15 isolates. Four IGS sequence types were found that included the two sequence types we identified in the ethanol-preserved ticks. The IGS sequences varied among the groups but there was not strict congruence. For example, IGS sequence type 1 was shared among all group A and four of the five group D spirochetes. Overall, three of the four groups of spirochetes (A, B, and C) were distinguished by their unique sequences and plasmid profiles. The fourth group (D) was distinct from the others but also displayed some heterogeneity in the *glpQ* and IGS sequences. The group D isolate DOS-6 was unique from all other isolates by its *glpQ* and IGS sequences, and its unique plasmid profile. Sixty-two borrelia DNA sequences have been deposited in GenBank with the following accession numbers: 16S rDNA (JX292896–JX292910); *flaB* (JX292911–JX292925); *glpQ* (JX292926–JX292940); IGS (JX292941–JX292957).

**Table 7:** Genomic groups, sequence types and plasmid types for 15 isolates of *Borrelia crocidurae* from Doucombo and Kalibombo, Mali.

Group	Isolate	16S rDNA	*flaB*	*glpQ*	IGS	Plasmid Type	Concatenate
A	DOU-690	1	1	1	1	I	X^a^
A	DOS-7	1	1	1	1	I	
A	DOS-5	1	1	1	1	I	
A	DOU-1b	1	1	1	1	I	
A	KOS-39	1	1	1	1	I	
B	DOS-2	2	2	2	2	II	X
B	DOS-27	2	2	2	2	II	
B	DOS-56	2	2	2	2	II	
C	DOS-3	3	3	3	3	III	X
C	DOS-13	3	3	3	3	III	
D	DOU	4	4	4	1	IV	X
D	DOU-686	4	4	4	1	IV	
D	DOS-16	4	4	4	1	IV	
D	KOS-46	4	4	5	1	V	X
D	DOS-6	4	4	2	4	VI	X

a X; the concatenated sequence for this isolate included in Figure 6.

**Figure 5:**
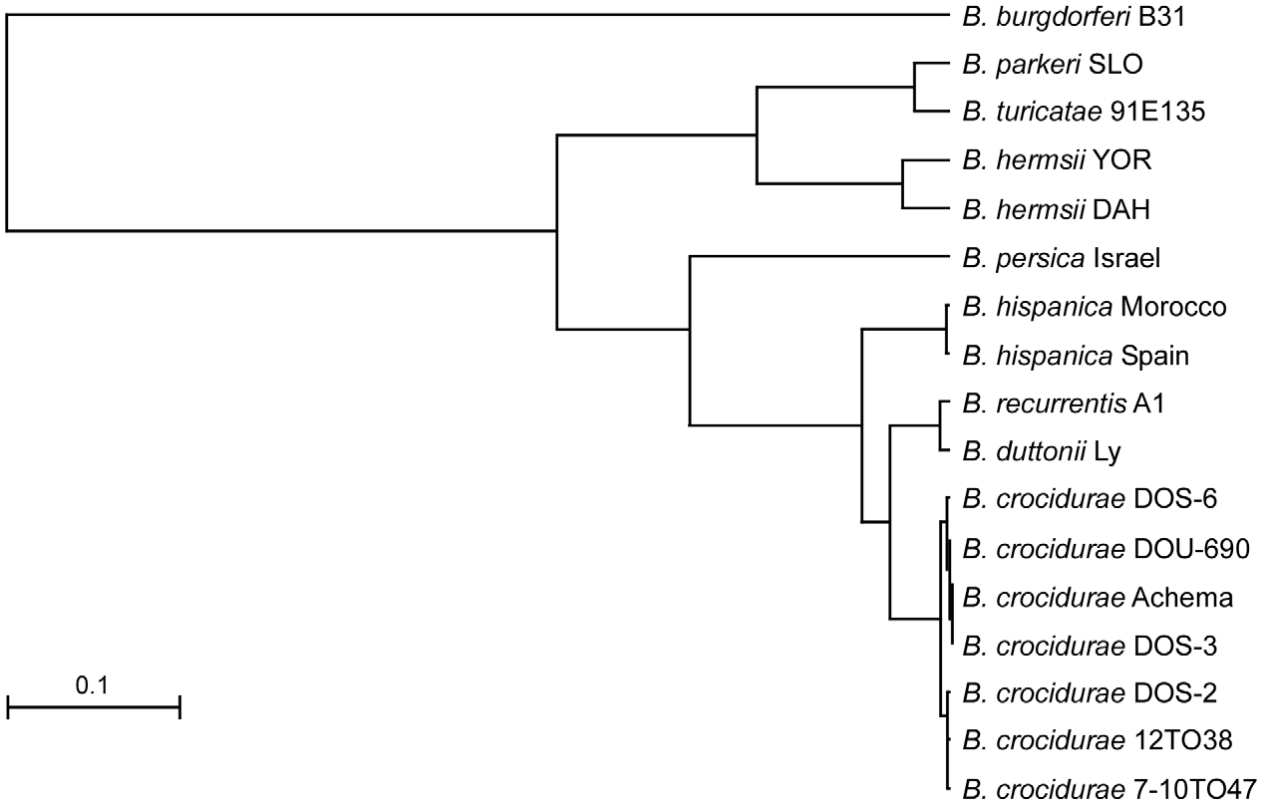
Phylogram based on the intergenic spacer sequences (IGS) for relapsing fever spirochetes. The Malian isolates, represented by the four IGS sequence types, group with *Borrelia crocidurae* from Mauritania (Achema) and Tunisia (#12T038 and #7-10T047). *Borrelia burgdorferi* B31 is the out-group. The scale bar represents the number of base substitutions per nucleotide.

**Figure 6:**
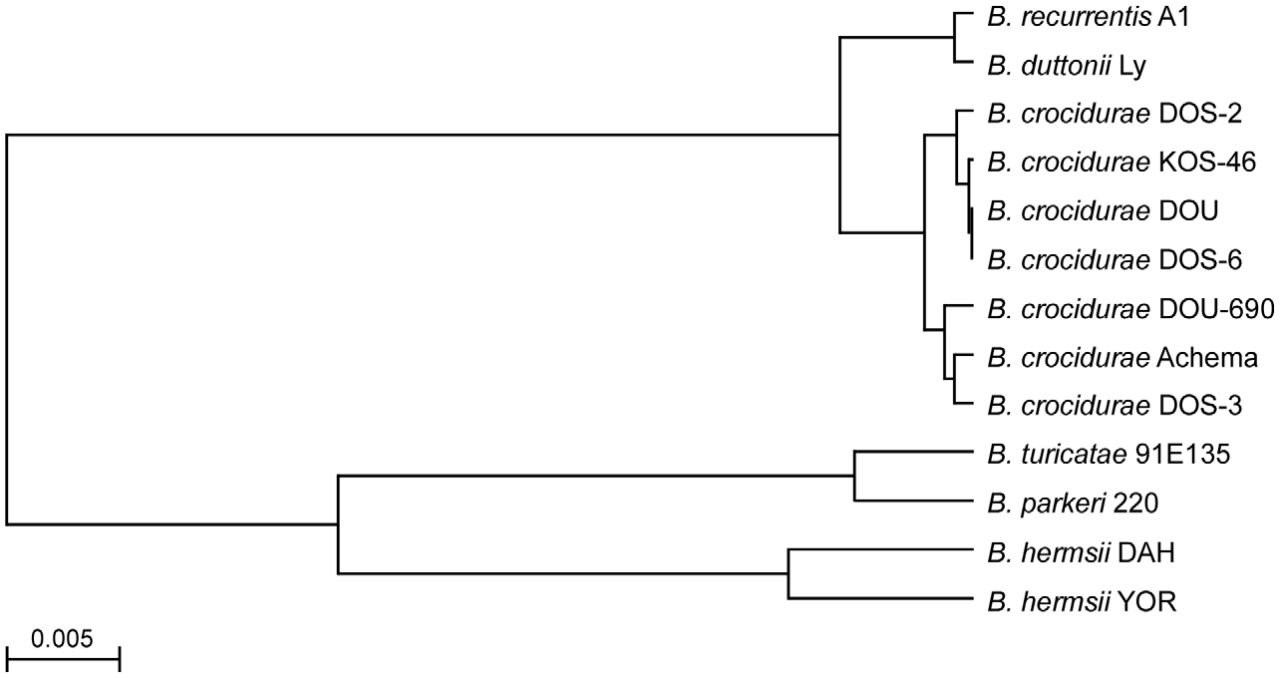
Phylogram based on the concatenated DNA sequences of the 16S rDNA, *flaB* and *glpQ* loci. The Malian isolates, represented by the six unique sequence types, group with *Borrelia crocidurae* from Mauritania (Achema). Scale bar represents the number of base substitutions per nucleotide.

Phylograms derived from multiple alignments for each locus including our isolates and sequences in the database all grouped the Malian spirochetes with *B. crocidurae* (data not shown), with one exception. The partial DNA sequence of the 16S rDNA (1,262 bp) for isolate DOS-2 was identical to the same length of sequence for *B. duttonii* Ly. We present two phylograms based on the IGS locus (Figure 5) and the concatenated sequence comprised of the 16S rDNA (1,262 bp), *flaB* (990 bp), and *glpQ* (1,002 bp) (3,254 bp total) (Figure 6). The four unique IGS sequences from all our Malian *B. crocidurae* aligned closest with IGS sequences for *B. crocidurae* from Mauritania (Achema strain) and Tunisia (7-10TO47 and 12TO38 DNA samples from infected ticks). This locus clearly grouped the *B. crocidurae* samples and distinguished them from the other Old World species (*B. duttonii, B. recurrentis, B. persica and B. hispanica*) (Figure 5). The percentage identity values for the IGS sequences among the seven *B. crocidurae* samples in heterologous matches ranged from 98.2 to 99.8% but these sequences had identity values of 65 to 91.9% when compared to the other Old World species (Table S3). The identity values were considerably less (49.7 to 57.3%) when the *B. crocidurae* samples were compared to the New World relapsing fever spirochete species *Borrelia hermsii*, *Borrelia turicatae*, and *Borrelia parkeri*. The phylogram constructed with the concatenated sequences (Figure 6) contained fewer Old World species of *Borrelia* than did the IGS analysis (Figure 5), because fewer sequences were available. We included the Achema strain of *B. crocidurae* because complete sequences were available for the three loci (CP003465) [53]. This analysis again identified the Malian spirochetes as *B. crocidurae*, which were clearly separated from the two very closely related species *B. duttonii* and *B. recurrentis*. Percentage identity values among the 15 Malian isolates (six unique sequences) were high at 99.5 to 99.9% (Table S4).

## Discussion

One of our primary methods to determine the presence of relapsing fever spirochetes in the small mammals was done indirectly with serological tests for anti-relapsing fever spirochete antibodies that identified animals that had been previously infected. Serological approaches for the surveillance of other vector-borne pathogens have been used for many years, such as wild carnivore serology for plague [54] and the use of sentinel chickens to monitor seroconversion for seasonal activity of numerous mosquito-borne viruses [55]. Serological surveys for relapsing fever have rarely been used for field studies [56], [57] and have never been utilized for the studies of enzootic foci of relapsing fever in Senegal or other regions of Africa. Earlier concerns for the specificity of such tests for relapsing fever antibodies were directed at the extreme antigenic variation known for these bacteria during infection [58] (hence what antigens should or could be used), and the antigenic relatedness among different *Borrelia* species, which results in the lack of specificity of the antibodies detected [59]. Such serological cross reactivity meant that people having antibodies reactive to antigens of *Borrelia burgdorferi*, the cause of Lyme borreliosis, may have actually been infected with relapsing fever spirochetes, with the reverse also being true. This dilemma was rectified to a large extent with the identification of an immunogenic protein in the relapsing fever spirochetes, glycerophosphodiester phosphodiesterase (GlpQ), which is absent in the agents of Lyme borreliosis [42], [43], [60]. For studies in North America where both Lyme borreliosis and relapsing fever exist, the application of GlpQ has helped to serologically discriminate people and wild mammals that were infected previously with relapsing fever spirochetes and not *B. burgdorferi*. While the presence of Lyme borreliosis spirochetes throughout West Africa is unknown and doubtful given the ecological requirement of the *Ixodes* species of ticks [61], our test would discriminate between such prior infections.

The strength of a specific and sensitive serological test for relapsing fever compared to a blood smear taken from the same animal lies in the temporal persistence of antibodies after infection compared to the brief and transient time when spirochetes are detectable in the blood. Therefore, in a population or community of animals susceptible to a bacterial infection, the proportion of individuals that are seropositive should increase seasonally whereas the number of animals actively infected at any one time may not. Our data demonstrate this utility of serology over active infection quite convincingly for spirochete activity. In the 20 villages we sampled, 14 villages had seropositive animals while only 5 villages had animals with detectable spirochetemias. These results were strengthened by the fact that for the six villages where no seropositive animals were found, neither was any animal found with active infection. Overall, 11.3% of the animals tested from all villages were seropositive while only 2.2% of the animals had spirochetes seen in their blood. Additionally, serology implicated eight species of mammals as hosts for spirochetes (Table 4) while the examination of blood smears found spirochetes in just five species (Table 6). The serological results were supported again by the blood smears as only those species that had seropositive individuals also had animals with spirochetes detected by microscopy.

We examined 50 microscopic fields for each blood smear to examine the mammals for spirochete infection. Investigators in Senegal typically examined 200 microscopic fields while looking for spirochetes in blood smears [18], [24], [26]. Therefore, our approach would have only a 25% chance at detecting a positive smear having only one spirochete in 200 fields, if the area of one microscope field and the volume of blood in each field were the same. However, as stated above we relied on serology to increase the sensitivity of our surveillance. We realize that microscopy is not the most sensitive method to detect relapsing fever spirochetes in the peripheral blood of an infected animal, and other investigators have on occasion used animal inoculation for studies on relapsing fever in Senegal. Diatta and colleagues examined 82 rodents comprised of three species collected in Dielmo, Senegal, and compared the success of examining blood smears to inoculating their blood and brain suspensions in laboratory mice to detect infection in the wild animals [24]. *Mastomys erythroleucus* comprised 89% (73 of 82 total) of the animals examined and from them only one blood slide was positive while five of the blood samples produced spirochetemias in mice, and brain tissues from 10 animals yielded laboratory infections. Thus brain inoculations were ten-times more sensitive at detecting infections in the wild rats compared to the microscopic examination of stained blood smears. More recently, Vial and colleagues expanded the studies in the same village in Senegal, and again they found that the inoculation of brain suspensions from the wild mammals into laboratory mice resulted in 12% infection compared to only 0.74% prevalence of infection based on the examination of blood smears [18]. Nordstand and colleagues also detected *B. crocidurae* and *B. duttonii* in patient blood samples by PCR when no spirochetes were observed by microscopy [31]. We probably missed some active infections in the animals we captured by examining only 50 microscopic fields and not utilizing PCR. We relied on serological surveillance to complement our microscopic examinations to increase our ability to detect the presence of spirochetes circulating in the numerous locations and species we sampled.

Our efforts across southern Mali demonstrated that many of the villages had spirochetes infecting several species of rodents and shrews, however the prevalence of infection was low. We began searching for ticks in a few villages that had higher seroprevalence rates, such as Belenikegny on the Bani River. Our initial attempts to find *O. sonrai* ticks there were unsuccessful. Then in late September 2010, nearly the entire village was flooded when the Bani River overflowed its banks. We redirected our efforts to the Bandiagara region, where our attempts to find ticks were successful. Additional trapping of the small mammals there demonstrated that in two nearby villages, Doucombo and Kalibombo, 35% of the rodents and shrews were seropositive and 10% of the animals had positive blood smears at the time of capture. The spirochetemias in some of the animals were quite high (Table 6) with three of the infected *M. natalensis* having 112, 140, and 264 spirochetes observed in the 50 fields examined. The studies in Senegal did not report the numbers of spirochetes seen in the blood of wild mammals, although for clinical investigations with human blood the densities were low; 75% of the smears contained less than 20 spirochetes in 200 fields [23].

*Ornithodoros sonrai* ticks were difficult to find until we intensified our efforts at Doucombo and Kalibombo. Here, the ticks were abundant and present in burrows in most of the houses we sampled. Ticks had a prevalence of spirochete infection of 17–18%. The estimates of infection were strikingly similar based on the PCR assays of alcohol-preserved ticks and when live ticks were fed on mice and transmitted spirochetes to them. In and around Dielmo, Senegal, the prevalence of *B. crocidurae* infection in *O. sonrai* ticks varied between 21 to 66%, based on PCR and DNA sequencing the *flaB* gene [18]. In transect surveys in Senegal, Mauritania and Mali, *O. sonrai* ticks were found in 26 of 30 villages sampled [18], although the publication does not specifically state what was found in Mali. However, Trape reported elsewhere that *O. sonrai* ticks were found in burrows in Djougounte, Sama, Molibana and Gao, Mali [27]. A survey of small mammal burrows in Tunisia found 15.1% of the *O. erraticus* ticks were infected with borrelia [16]. The uncultured spirochetes were identified as *B. crocidurae* by sequencing the DNA of amplicons of the 16S rDNA, *flaB*, and IGS loci. Much earlier surveys in Egypt, which predated the development of PCR and a culture medium for borrelia, demonstrated that 76 of 215 pools of *O. erraticus* ticks collected from rodent burrows transmitted spirochetes, assumed to be *B. crocidurae*, when fed on laboratory mice [17].

We established 15 novel *in vitro* isolates of *B. crocidurae* from Malian ticks and rodents. These isolates allowed us to get a preliminary characterization of the genetic diversity from this group of spirochetes, and to compare our molecular data to the results of previous investigations. However, while reviewing the literature we soon realized that very few *in vitro* cultures of *B. crocidurae* existed prior to our work. van Dam and colleagues claimed to be the first to isolate *B. crocidurae* in culture from the blood of two patients infected in The Gambia and Senegal in 1997 [62]. Yet, three years earlier Fukunaga and colleagues included two strains of *B. crocidurae* (ORI and one isolate not designated) in their phylogenetic analysis of *Borrelia* species that had been grown in BSK-II medium [63]. Previous work to characterize Old World relapsing fever borrelia utilized spirochetes that were isolated and maintained by serial passage in mice [28], [64]. Ras and colleagues performed the first large scale phylogenetic analysis of what they called “noncultivatable” relapsing fever spirochetes by utilizing PCR to amplify the 16S rDNA from spirochetes in the blood of infected laboratory mice [64]. Their analysis included nine *in vivo* isolates of *B. crocidurae* that originated from ticks, human blood, and rodents from Mauritania, Senegal, Morocco and Mali. The two *in vivo* isolates from Mali, BAR and SIS, were those spirochetes from human patients infected in 1977 and 1988, first reported by Rodchain et al. [28]. In spite of the various locations and biological sources for the nine *in vivo* isolates of *B. crocidurae* examined by Ras and colleagues, the 16S rDNA sequences were identical [64]. This is in contrast to what we found among our 15 isolates of *B. crocidurae* from Doucombo and Kalibombo. At these two nearby villages, we identified four 16S rDNA sequences, one of which (from isolate DOS-2) was identical to the sequence for the Achema strain of *B. crocidurae* studied by Ras and colleagues [64] and two other research groups [53], [65].

During the epidemiological investigations of tick-borne relapsing fever in Senegal, no attempts were made to culture or identify the spirochetes observed in humans, wild mammals or ticks [23]–[26]. Naming the spirochetes as *B. crocidurae* during these studies was based on the identity of the tick vector, *O. sonrai*, which is not known to transmit any other species of relapsing fever spirochete. More recently, Trape and his colleagues reported the incidence of human relapsing fever in Dielmo, Senegal, for 14 consecutive years (1990–2003) [18]. Small mammals and ticks were also collected and examined for spirochete infection. Spirochetes detected in human blood and small mammals were not isolated or identified but *O. sonrai* ticks were assayed by PCR. Partial internal fragments of *flaB* were sequenced from infected *O. sonrai* ticks collected in Senegal and Mauritania [18], [22]. All sequences (number of samples not stated) were identical but the one partial sequence deposited (284 bp; DQ234749) varied by 1 bp from our *flaB* sequences within the 284 bp that could be compared.

The trend in recent years to identify *B. crocidurae* has been to use as little sequence data as possible via PCR using one or more partial coding or non-coding targets. Most of these approaches have been used to identify spirochetes in people living in or having traveled to endemic areas of West Africa [29], [66]–[70]. The clinical diagnostic approach has merit but eliminates the potential to gain more genetic and biological information had these spirochetes been isolated in culture. For example, through the many efforts of Cutler and her collaborators, the borrelia research arena has benefited tremendously by having many isolates of *B. recurrentis* and *B. duttonii* established *in vitro* [71]–[74]. These isolates have provided the basis for a greater understanding of the genetic diversity and molecular biology of African relapsing fever spirochetes, and they provided the material for a whole genome comparison of these two important louse- and tick-borne pathogens [53].

Our isolates of *B. crocidurae* demonstrated a rather striking amount of genetic diversity in the plasmid content and DNA sequences of highly conserved genes. In 1986, Hyde and Johnson first reported that *B. crocidurae* harbored plasmids [75]. However, we found nothing in the literature to which we could compare our findings, which is the variation in number and size of plasmids among different isolates. The genome of the Achema strain of *B. crocidurae* has been determined (CP003426–CP003465) [53], although the plasmid-associated contigs were not assembled into their full-length native molecules. Our estimate of at least 10 linear and one or more circular plasmids in our isolates of *B. crocidurae* may be an underestimate. *B. duttonii* Ly and *B. recurrentis* A1 contain 16 and 7 plasmids, respectively [53], although the number and size of plasmids varies among isolates for both species [72], [73], as we observed for *B. crocidurae* (Figure 4).

Our MLST method identified the spirochete isolates as *B. crocidurae* and identified four distinct genomic groups. We and other collaborators have used this approach to characterize the North American relapsing fever spirochetes *B. hermsii*, *B. turicatae* and *B. parkeri* [49], [76], [77]. Toledo and colleagues applied MLST to identify an isolate of relapsing fever spirochete from Spain as *Borrelia hispanica* [65]. Recently, the chromosomes of *B. recurrentis* A1, *B. duttonii* Ly and *B. crocidurae* Achema were aligned to identify homologous non-coding, intergenic spacer sequences (not the IGS locus) that were used to develop a PCR – DNA sequence typing scheme to distinguish these three species of spirochetes [53]. This multispacer sequence typing utilized the concatenated sequence of five intergenic spacers that totaled approximately 2,300 bp. The method was applied to 60 samples of infected human blood from relapsing fever patients from Ethiopia (30 *B. recurrentis* samples), Tanzania (17 *B. duttonii* samples) and Senegal (13 *B. crocidurae* samples). The method clearly distinguished the three species of spirochetes with no overlap, which was not the case when using the IGS region [78], [79]. The method also demonstrated seven types among the 13 samples of *B. crocidurae* compared to only five types among the 47 samples representing the other two species [53]. Clearly, a pattern is emerging from our efforts and those of Elbir and colleagues [53] that shows a much more diverse population structure for *B. crocidurae*, an enzootic pathogen with multiple vertebrate hosts, than has yet been demonstrated for either *B. recurrentis* or *B. duttonii*, neither of which has a nonhuman vertebrate host.

Herein we present evidence for the widespread occurrence of relapsing fever spirochetes infecting small mammals across southern Mali. In the Bandiagara area, we identified two villages where the essential triangle for an enzootic focus for a vector-borne disease exists. The susceptible peridomestic rodents and shrews, the tick vector *O. sonrai*, and the spirochetal agent *B. crocidurae* all coexist with the human population. Therefore, we conclude that the potential for human infections is present in Mali, as has been described in Senegal [18]. During the course of our studies, a young girl who visited Mopti, approximately 75 km west-northwest of Bandiagara, was diagnosed with a *B. crocidurae* infection when she returned to France [29]. While her blood smears were negative for plasmodia, spirochetes were eventually detected retrospectively after antibiotic treatment. Just as it was over 100 years ago and remains today, distinguishing tick-borne relapsing fever from human malaria remains a clinical and diagnostic challenge [2], [31]. We hope that our efforts described herein will stimulate future investigations to determine the extent to which tick-borne relapsing fever is a human public health problem in Mali.

## Supporting Information

Table S1 **The villages in Mali investigated, dates, species and number of animals captured, number of serological tests performed, and the number of animals seropositive.**
(DOC)

Table S2 **Mammal skulls from Mali deposited as voucher specimens in the Smithsonian Institution Division of Mammals Collection and GenBank accession numbers for mt** ***cyt-b*** **sequences.**
(DOC)

Table S3 **DNA sequence percentage identity values for the IGS locus.**
(DOC)

Table S4 **DNA sequence identity values for concatenated sequences comprised of the 16S rDNA,** ***flaB*** **and** ***glpQ*** **loci.**
(DOC)
