# Supplementary material for: Endemic Foci of the Tick-Borne Relapsing Fever Spirochete Borrelia crocidurae in Mali, West Africa, and the Potential for Human Infection
Source: PLoS Negl Trop Dis. 2012 Nov 29;6(11):e1924. doi: 10.1371/journal.pntd.0001924 (PMC3510061; doi:10.1371/journal.pntd.0001924)
Supplement: Table S1 — The villages in Mali investigated, dates, species and number of animals captured, number of serological tests performed, and the number of animals seropositive. (DOC) [file pntd.0001924.s001.doc]

**Table S1.** The villages in Mali investigated, dates, species and number of animals captured, number of serological tests performed, and the number of animals seropositive.

| **Village** | **Date - Species** | **#Captured** | **#Tested** | **#Positive** |
| --- | --- | --- | --- | --- |
| Molibana | 6 December 2007 |  |  |  |
|  | *Mastomys natalensis* | 1 | 1 | 1 |
|  | *Mastomys erythroleucus* | 12 | 12 | 5 |
|  | *Praomys daltoni* | 3 | 2 | 0 |
|  | *Arvicanthis niloticus* | 7 | 7 | 0 |
|  | *Taterillus gracilis* | 1 | 1 | 0 |
| Sama | 7 December 2007 |  |  |  |
|  | *Arvicanthis niloticus* | 1 | 1 | 1 |
|  | *Taterillus gracilis* | 3 | 3 | 0 |
|  | *Crocidura viaria* | 1 | 1 | 1 |
|  | *Crocidura fulvastra* | 1 | 1 | 1 |
| Sefeto West | 9 December 2007 |  |  |  |
|  | *Praomys daltoni* | 2 | 2 | 0 |
|  | *Crocidura olivieri* | 1 | 1 | 1 |
| Djougounte | 10 December 2007 |  |  |  |
|  | *Mastomys erythroleucus* | 1 | 1 | 0 |
|  | *Praomys daltoni* | 3 | 3 | 1 |
|  | *Crocidura olivieri* | 3 | 3 | 1 |
|  | *Crocidura fulvastra* | 2 | 2 | 0 |
| Djidian | 11 December 2007 |  |  |  |
|  | *Mastomys natalensis* | 10 | 10 | 0 |
|  | *Praomys daltoni* | 8 | 8 | 0 |
|  | *Crocidura olivieri* | 1 | 1 | 0 |
| Bozokin | 12 January 2009 |  |  |  |
|  | *Mastomys natalensis* | 19 | 19 | 0 |
| Kenieroba | 13 January 2009 |  |  |  |
|  | *Mastomys natalensis* | 8 | 7 | 0 |
|  | *Mastomys erythroleucus* | 4 | 3 | 0 |
|  | *Crocidura olivieri* | 8 | 8 | 1 |
| Fourda | 14 January 2009 |  |  |  |
|  | *Mastomys natalensis* | 14 | 14 | 0 |
|  | *Crocidura olivieri* | 3 | 3 | 1 |
| Sinkerma | 17 January 2009 |  |  |  |
|  | *Mastomys natalensis* | 3 | 3 | 1 |
|  | *Praomys daltoni* | 7 | 7 | 2 |
|  | *Acomys airensis* | 4 | 4 | 0 |
|  | *Gerbillus campestris* | 1 | 1 | 0 |
|  | *Crocidura sp*. | 1 | 1 | 0 |
| Petaka | 18 January 2009 |  |  |  |
|  | *Mastomys natalensis* | 6 | 6 | 0 |
|  | *Mastomys erythroleucus* | 8 | 8 | 0 |
|  | *Praomys daltoni* | 16 | 16 | 1 |
|  | *Arvicanthis niloticus* | 4 | 4 | 0 |
|  | *Taterillus gracilis* | 2 | 2 | 0 |
|  | *Mus musculoides* | 1 | 1 | 0 |
|  | *Crocidura viaria* | 2 | 2 | 0 |
| Belenikegny | 19 January 2009 |  |  |  |
|  | *Mastomys natalensis* | 1 | 1 | 0 |
|  | *Mastomys erythroleucus* | 11 | 11 | 2 |
|  | *Mastomys huberti* | 3 | 3 | 1 |
|  | *Arvicanthis niloticus* | 1 | 1 | 0 |
|  | *Rattus rattus* | 6 | 6 | 0 |
|  | *Crocidura olivieri* | 5 | 5 | 0 |
| N’Tessoni | 5 -6 June 2009 |  |  |  |
|  | *Mastomys natalensis* | 15 | 15 | 0 |
|  | *Mastomys erythroleucus* | 2 | 2 | 0 |
|  | *Praomys daltoni* | 1 | 1 | 0 |
|  | *Arvicanthis niloticus* | 1 | 1 | 0 |
|  | *Mus musculoides* | 3 | 1 | 0 |
|  | *Crocidura olivieri* | 3 | 3 | 0 |
| Soromba | 8 June 2009 |  |  |  |
|  | *Mastomys natalensis* | 25 | 25 | 0 |
| Doneguebougou | 12-14 June 2009 |  |  |  |
|  | *Mastomys natalensis* | 38 | 38 | 1 |
|  | *Mastomys erythroleucus* | 2 | 2 | 0 |
|  | *Praomys daltoni* | 1 | 1 | 0 |
|  | *Arvicanthis niloticus* | 1 | 1 | 0 |
|  | *Rattus rattus* | 1 | 1 | 0 |
|  | *Crocidura olivieri* | 7 | 7 | 0 |
|  | *Crocidura viaria* | 3 | 3 | 0 |
| Belenikegny | 5-6 January 2010 |  |  |  |
|  | *Mastomys natalensis* | 14 | 14 | 1 |
|  | *Mastomys erythroleucus* | 39 | 38 | 5 |
|  | *Mastomys huberti* | 36 | 35 | 4 |
|  | *Rattus rattus* | 13 | 13 | 0 |
|  | *Crocidura olivieri* | 9 | 9 | 0 |
| Soromba | 8-9 January 2010 |  |  |  |
|  | *Mastomys natalensis* | 21 | 21 | 2 |
| Komina | 8-9 January 2010 |  |  |  |
|  | *Mastomys natalensis* | 12 | 12 | 2 |
| Belenikegny | 3-5 September 2010 |  |  |  |
|  | *Mastomys natalensis* | 27 | 26 | 1 |
|  | *Mastomys erythroleucus* | 1 | 1 | 0 |
|  | *Mastomys huberti* | 16 | 16 | 0 |
|  | *Arvicanthis niloticus* | 1 | 1 | 0 |
|  | *Rattus rattus* | 9 | 9 | 0 |
|  | *Taterillus gracilis* | 1 | 1 | 0 |
|  | *Crocidura olivieri* | 5 | 5 | 0 |
| Kalibombo | 23 April 2011 |  |  |  |
|  | *Mastomys natalensis* | 14 | 13 | 3 |
|  | *Praomys daltoni* | 1 | 1 | 0 |
|  | *Crocidura olivieri* | 4 | 3 | 1 |
| Doucombo | 24-25 April 2011 |  |  |  |
|  | *Mastomys natalensis* | 24 | 19 | 10 |
|  | *Praomys daltoni* | 1 | 1 | 0 |
|  | *Acomys airensis* | 1 | 1 | 0 |
|  | *Crocidura olivieri* | 7 | 4 | 2 |
| Doneguebougou | 24-26 September 2011 |  |  |  |
|  | *Mastomys natalensis* | 41 | 41 | 0 |
| Doucombo | 30 September & 2 October 2011 |  |  |  |
|  | *Mastomys natalensis* | 46 | 46 | 18 |
|  | *Praomys daltoni* | 5 | 5 | 2 |
| Kalibombo | 1 October 2011 |  |  |  |
|  | *Mastomys natalensis* | 33 | 33 | 7 |
|  | *Praomys daltoni* | 2 | 2 | 2 |
| Kerekoumala | 5 October 2011 |  |  |  |
|  | *Mastomys natalensis* | 26 | 26 | 0 |
| Garalo | 5-6 October 2011 |  |  |  |
|  | *Mastomys natalensis* | 22 | 22 | 0 |
| Kotie’ | 6 October 2011 |  |  |  |
|  | *Mastomys natalensis* | 11 | 11 | 0 |
| **Totals** |  | **744** | **726** | **82** |
