# Supplementary material for: Endemic Foci of the Tick-Borne Relapsing Fever Spirochete Borrelia crocidurae in Mali, West Africa, and the Potential for Human Infection
Source: PLoS Negl Trop Dis. 2012 Nov 29;6(11):e1924. doi: 10.1371/journal.pntd.0001924 (PMC3510061; doi:10.1371/journal.pntd.0001924)
Supplement: Table S2 — Mammal skulls from Mali deposited as voucher specimens in the Smithsonian Institution Division of Mammals Collection and GenBank accession numbers for mt cyt-b sequences. (DOC) [file pntd.0001924.s002.doc]

**Table S2.** Mammal skulls from Mali deposited as voucher specimens in the Smithsonian Institution Division of Mammals Collection and GenBank accession numbers for mt *cyt-b* sequences.

|  |  |  |  |  |  |
| --- | --- | --- | --- | --- | --- |
| **Number** | **Species and Sex** | **Weight (g)** | **Date Collected** | **Village** | **GenBank #** |
| M#-168 | *Mastomys natalensis* **♀** | 72 | 18 January 2009 | Petaka | JX292854 |
| M#-173 | *Mastomys natalensis* ♀ | 52 | 18 January 2009 | Petaka | JX292885 |
| M#-210 | *Mastomys natalensis* ♀ | 50 | 5 June 2009 | N’Tessoni | JX292876 |
| M#-211 | *Mastomys natalensis* ♀ | 68 | 5 June 2009 | N’Tessoni | JX292860 |
| M#-213 | *Mastomys natalensis* ♀ | 48 | 5 June 2009 | N’Tessoni |  |
| M#-260 | *Mastomys natalensis* ♂ | 30 | 12 June 2009 | Doneguebougou |  |
| M#-261 | *Mastomys natalensis* ♀ | 23 | 12 June 2009 | Doneguebougou |  |
| M#-262 | *Mastomys natalensis* ♀ | 50 | 12 June 2009 | Doneguebougou |  |
| M#-265 | *Mastomys natalensis* ♀ | 80 | 12 June 2009 | Doneguebougou | JX292861 |
| M#-266 | *Mastomys natalensis* ♂ | 20 | 12 June 2009 | Doneguebougou |  |
| M#-281 | *Mastomys natalensis* ♂ | 75 | 13 June 2009 | Doneguebougou | JX292862 |
| M#-282 | *Mastomys natalensis* ♂ | 55 | 13 June 2009 | Doneguebougou |  |
| M#-285 | *Mastomys natalensis* ♀ | 45 | 13 June 2009 | Doneguebougou |  |
| M#-403 | *Mastomys natalensis* ♂ | 70 | 6 January 2010 | Belenikegny | JX292865 |
| M#-406 | *Mastomys natalensis* ♀ | 85 | 6 January 2010 | Belenikegny | JX292866 |
| M#-415 | *Mastomys natalensis* ♂ | 78 | 6 January 2010 | Belenikegny |  |
| M#-558 | *Mastomys natalensis* ♀ | 70 | 25 April 2011 | Doucombo | JX292882 |
| M#-562 | *Mastomys natalensis* ♂ | 40 | 25 April 2011 | Doucombo |  |
| M#-568 | *Mastomys natalensis* ♂ | 72 | 25 April 2011 | Doucombo | JX292883 |
| M#-274 | *Mastomys erythroleucus* ♀ | 40 | 12 June 2009 | Doneguebougou | JX292877 |
| M#-275 | *Mastomys erythroleucus* ♂ | 47 | 12 June 2009 | Doneguebougou | JX292863 |
| M#-323 | *Mastomys erythroleucus* ♂ | 18 | 5 January 2010 | Belenikegny |  |
| M#-330 | *Mastomys erythroleucus* ♀ | 24 | 5 January 2010 | Belenikegny |  |
| M#-335 | *Mastomys erythroleucus* ♂ | 23 | 5 January 2010 | Belenikegny |  |
| M#-339 | *Mastomys erythroleucus* ♀ | 19 | 5 January 2010 | Belenikegny |  |
| M#-341 | *Mastomys erythroleucus* ♂ | 23 | 5 January 2010 | Belenikegny |  |
| M#-342 | *Mastomys erythroleucus* ♂ | 46 | 5 January 2010 | Belenikegny |  |
| M#-377 | *Mastomys erythroleucus* ♀ | 58 | 6 January 2010 | Belenikegny | JX292867 |
| M#-388 | *Mastomys erythroleucus* ♂ | 48 | 6 January 2010 | Belenikegny | JX292868 |
| M#-393 | *Mastomys erythroleucus* ♀ | 37 | 6 January 2010 | Belenikegny |  |
| M#-459 | *Mastomys erythroleucus* ♀ | 47 | 3 September 2010 | Belenikegny |  |
| M#-343 | *Mastomys huberti* ♀ | 27 | 5 January 2010 | Belenikegny |  |
| M#-368 | *Mastomys huberti* ♂ | 36 | 6 January 2010 | Belenikegny |  |
| M#-387 | *Mastomys huberti* ♀ | 19 | 6 January 2010 | Belenikegny |  |
| M#-475 | *Mastomys huberti* ♂ | 70 | 4 September 2010 | Belenikegny |  |
| M#-477 | *Mastomys huberti* ♂ | 36 | 4 September 2010 | Belenikegny |  |
| M#-481 | *Mastomys huberti* ♂ | 55 | 4 September 2010 | Belenikegny | JX292873 |
| M#-483 | *Mastomys huberti* ♂ | 77 | 4 September 2010 | Belenikegny | JX292874 |
| M#-486 | *Mastomys huberti* ♂ | 58 | 4 September 2010 | Belenikegny |  |
| M#-489 | *Mastomys huberti* ♂ | 50 | 4 September 2010 | Belenikegny |  |
| M#-216 | *Praomys daltoni* ♀ | 32 | 5 June 2009 | N’Tessoni | JX292891 |
| M#-263 | *Praomys daltoni* ♀ | 28 | 12 June 2009 | Doneguebougou | JX292895 |
| M#-533 | *Praomys daltoni* ♀ | 29 | 23 April 2011 | Kalibombo | JX292871 |
| M#-543 | *Praomys daltoni* ♂ | 23 | 24 April 2011 | Doucombo | JX292881 |
| M#-277 | *Rattus rattus* ♀ | 109 | 12 June 2009 | Doneguebougou | JX292864 |
| M#-347 | *Rattus rattus* ♂ | 50 | 5 January 2010 | Belenikegny |  |
| M#-348 | *Rattus rattus* ♂ | 21 | 5 January 2010 | Belenikegny |  |
| M#-361 | *Rattus rattus* ♂ | 140 | 5 January 2010 | Belenikegny | JX292879 |
| M#-404 | *Rattus rattus* ♀ | 42 | 6 January 2010 | Belenikegny |  |
| M#-405 | *Rattus rattus* ♂ | 38 | 6 January 2010 | Belenikegny |  |
| M#-465 | *Rattus rattus* ♂ | 80 | 3 September 2010 | Belenikegny |  |
| M#-498 | *Rattus rattus* ♀ | 129 | 4 September 2010 | Belenikegny | JX292875 |
| M#-221 | *Arvicanthis niloticus* ♀ | 93 | 5 June 2009 | N’Tessoni | JX292886 |
| M#-276 | *Arvicanthis niloticus* ♂ | 135 | 12 June 2009 | Doneguebougou |  |
| M#-458 | *Arvicanthis niloticus* ♀ | 100 | 3 September 2010 | Belenikegny | JX292872 |
| M#-457 | *Taterillus gracilis* ♂ | 57 | 3 September 2010 | Belenikegny |  |
| M#-550 | *Acomys airensis* ♀ | 40 | 24 April 2011 | Doucombo | JX292880 |
| M#-219 | *Crocidura olivieri* ♀ | 45 | 5 June 2009 | N’Tessoni | JX292878 |
| M#-353 | *Crocidura olivieri* ♀ | 59 | 5 January 2010 | Belenikegny | JX292869 |
| M#-401 | *Crocidura olivieri* ♂ | 67 | 6 January 2010 | Belenikegny | JX292870 |
