# Supplementary material for: Endemic Foci of the Tick-Borne Relapsing Fever Spirochete Borrelia crocidurae in Mali, West Africa, and the Potential for Human Infection
Source: PLoS Negl Trop Dis. 2012 Nov 29;6(11):e1924. doi: 10.1371/journal.pntd.0001924 (PMC3510061; doi:10.1371/journal.pntd.0001924)
Supplement: Table S3 — DNA sequence percentage identity values for the IGS locus. (DOC) [file pntd.0001924.s003.doc]

| **Table S3**. DNA sequence percentage identity values for the IGS locus. |
| --- |

| Species and Isolate | B.burgdorferi B31 | B.hermsii DAH | B.hermsii YOR | B.turicatae 91E135 | B.parkeri SLO | B.recurrentis A1 | B. duttonii Ly | B.persica Israel | B.hispanica Spain | B.hispanica Morocco | B.crocidurae DOU-690 | B.crocidurae DOS-6 | B.crocidurae DOS-2 | B.crocidurae Achema | B.crocidurae 12TO38 | B.crocidurae 7-10TO47 | B.crocidurae DOS-3 |
| --- | --- | --- | --- | --- | --- | --- | --- | --- | --- | --- | --- | --- | --- | --- | --- | --- | --- |
| B.burgdorferi B31 | 100 |  |  |  |  |  |  |  |  |  |  |  |  |  |  |  |  |
| B.hermsii DAH | 34.7 | 100 |  |  |  |  |  |  |  |  |  |  |  |  |  |  |  |
| B.hermsii YOR | 34.1 | 94.2 | 100 |  |  |  |  |  |  |  |  |  |  |  |  |  |  |
| B.turicatae 91E135 | 38.6 | 76.1 | 76.8 | 100 |  |  |  |  |  |  |  |  |  |  |  |  |  |
| B.parkeri SLO | 35.7 | 69.2 | 69.9 | 82.7 | 100 |  |  |  |  |  |  |  |  |  |  |  |  |
| B.recurrentis A1 | 40.0 | 55.9 | 55.7 | 58.9 | 55.0 | 100 |  |  |  |  |  |  |  |  |  |  |  |
| B.duttonii Ly | 39.7 | 55.9 | 55.7 | 59.3 | 55.3 | 98.6 | 100 |  |  |  |  |  |  |  |  |  |  |
| B.persica Israel | 33.0 | 52.1 | 51.1 | 52.9 | 51.2 | 60.7 | 61.1 | 100 |  |  |  |  |  |  |  |  |  |
| B.hispanica Spain | 34.3 | 53.4 | 52.8 | 53.3 | 53.7 | 76.1 | 76.7 | 67.2 | 100 |  |  |  |  |  |  |  |  |
| B.hispanica Morocco | 34.1 | 53.0 | 52.4 | 53.0 | 53.3 | 75.9 | 76.5 | 67.2 | 99.3 | 100 |  |  |  |  |  |  |  |
| B.crocidurae DOU-690 | 39.9 | 56.7 | 55.2 | 58.8 | 55.8 | 91.1 | 91.5 | 65.0 | 79.3 | 79.5 | 100 |  |  |  |  |  |  |
| B.crocidurae DOS-6 | 39.6 | 56.9 | 55.4 | 58.8 | 55.8 | 91.5 | 91.9 | 65.6 | 79.7 | 79.9 | 99.2 | 100 |  |  |  |  |  |
| B.crocidurae DOS-2 | 39.9 | 56.7 | 55.4 | 58.8 | 55.8 | 91.1 | 91.7 | 65.6 | 79.9 | 80.1 | 98.2 | 99.0 | 100 |  |  |  |  |
| B.crocidurae Achema | 39.7 | 56.9 | 55.4 | 59.0 | 56.0 | 91.5 | 91.9 | 65.4 | 79.7 | 79.9 | 99.6 | 99.6 | 98.6 | 100 |  |  |  |
| B.crocidurae 12TO38 | 39.6 | 56.5 | 55.2 | 58.6 | 55.6 | 91.7 | 92.3 | 65.4 | 79.5 | 79.7 | 98.4 | 98.8 | 99.4 | 98.8 | 100 |  |  |
| B.crocidurae 7-10TO47 | 39.7 | 56.9 | 55.6 | 58.6 | 55.6 | 91.1 | 91.7 | 65.8 | 79.7 | 79.9 | 98.2 | 98.6 | 99.2 | 98.6 | 99.4 | 100 |  |
| B.crocidurae DOS-3 | 39.9 | 56.7 | 55.2 | 58.8 | 55.8 | 91.3 | 91.7 | 65.2 | 79.5 | 79.7 | 99.8 | 99.4 | 98.4 | 99.8 | 98.6 | 98.4 | 100 |
