# Supplementary material for: Endemic Foci of the Tick-Borne Relapsing Fever Spirochete Borrelia crocidurae in Mali, West Africa, and the Potential for Human Infection
Source: PLoS Negl Trop Dis. 2012 Nov 29;6(11):e1924. doi: 10.1371/journal.pntd.0001924 (PMC3510061; doi:10.1371/journal.pntd.0001924)
Supplement: Table S4 — DNA sequence identity values for concatenated sequences comprised of the 16S rDNA, flaB and glpQ loci. (DOC) [file pntd.0001924.s004.doc]

**Table S4.** DNA sequence identity values for concatenated sequences comprised of the 16S rDNA, *flaB* and *glpQ* loci.

| Species and Isolate | B.duttonii Ly | B.recurrentis A1 | B.crocidurae Achema | B.crocidurae KOS-46 | B.crocidurae DOU-690 | B.crocidurae DOU | B.crocidurae DOS-6 | B.crocidurae DOS-3 | B.crocidurae DOS-2 | B.turicatae 91E135 | B.parkeri 220 | B.hermsii DAH | B.hermsii YOR |
| --- | --- | --- | --- | --- | --- | --- | --- | --- | --- | --- | --- | --- | --- |
| B.duttonii Ly | 100.0 |  |  |  |  |  |  |  |  |  |  |  |  |
| B.recurrentis A1 | 99.8 | 100.0 |  |  |  |  |  |  |  |  |  |  |  |
| B.crocidurae Achema | 92.2 | 92.1 | 100.0 |  |  |  |  |  |  |  |  |  |  |
| B.crocidurae KOS-46 | 98.2 | 98.1 | 92.2 | 100.0 |  |  |  |  |  |  |  |  |  |
| B.crocidurae DOU-690 | 98.1 | 98.0 | 92.3 | 99.5 | 100.0 |  |  |  |  |  |  |  |  |
| B.crocidurae DOU | 98.2 | 98.1 | 92.2 | 99.9 | 99.6 | 100.0 |  |  |  |  |  |  |  |
| B.crocidurae DOS-6 | 98.2 | 98.1 | 92.2 | 99.9 | 99.6 | 99.9 | 100.0 |  |  |  |  |  |  |
| B.crocidurae DOS-3 | 98.2 | 98.0 | 92.3 | 99.5 | 99.7 | 99.5 | 99.5 | 100.0 |  |  |  |  |  |
| B.crocidurae DOS-2 | 98.3 | 98.2 | 92.3 | 99.8 | 99.5 | 99.8 | 99.9 | 99.6 | 100.0 |  |  |  |  |
| B.turicatae 91E135 | 90.2 | 90.2 | 84.9 | 90.9 | 90.8 | 91.0 | 91.0 | 90.9 | 91.0 | 100.0 |  |  |  |
| B.parkeri 220 | 90.3 | 90.2 | 84.9 | 91.0 | 90.9 | 91.0 | 91.0 | 90.9 | 91.1 | 98.9 | 100.0 |  |  |
| B.hermsii DAH | 90.4 | 90.3 | 85.3 | 90.4 | 90.2 | 90.4 | 90.4 | 90.2 | 90.3 | 93.5 | 93.4 | 100.0 |  |
| B.hermsii YOR | 90.2 | 90.2 | 85.1 | 90.3 | 90.0 | 90.3 | 90.3 | 90.0 | 90.2 | 93.5 | 93.3 | 98.3 | 100.0 |
